# Detection of Subclinical Synovial Inflammation by Microwave Radiometry

**DOI:** 10.1371/journal.pone.0064606

**Published:** 2013-05-31

**Authors:** Evangelia Zampeli, Ioannis Raftakis, Archontoula Michelongona, Chara Nikolaou, Antonia Elezoglou, Konstantinos Toutouzas, Elias Siores, Petros P. Sfikakis

**Affiliations:** 1 First Department of Propedeutic and Internal Medicine, Athens University Medical School, Athens, Greece; 2 Rheumatology Department, Asklepion General Hospital, Athens, Greece; 3 First Department of Cardiology, Athens University Medical School, Athens, Greece; 4 Institute for Materials Research and Innovation, University of Bolton, Bolton, United Kingdom; University of Patras Medical School, Greece

## Abstract

**Objective:**

Microwave Radiometry is a non-invasive method which determines within seconds the *in vivo* temperature of internal tissues at a depth of 3–7 cm with an accuracy of ±0.2°C. In this proof-of-concept study, we tested the hypothesis that, in absence of relevant clinical signs, increased local temperature detected by microwave radiometry reflects subclinical synovial inflammation, using ultrasound as reference method.

**Methods:**

Knees of healthy controls, subjects with recent knee trauma and symptom-free patients with rheumatoid arthritis (RA) or osteoarthritis were examined by placing the microwave radiometry sensor, a) at the upper one third of the anterior surface of the thigh (control-point), and b) over the suprapatellar recess. Ultrasound was performed immediately after and the possible presence of fluid and/or synovitis was correlated with microwave radiometry findings.

**Results:**

In 30 healthy and 10 injured knees the temperature was always lower than thigh (32.3±1.1 and 31.8±1.4 *versus* 34.1±0.9 and 33.6±1.2°C with a difference (ΔΤ) of −1.8±0.2 and −1.9±0.4°C respectively). Of 40 RA and 20 osteoarthritis knees examined, ultrasound findings indicative of subclinical inflammation (fluid effusion and/or Doppler signal) were found in 24 and 12, respectively, in which the temperature was higher than healthy knees and ΔΤ was lower (−0.9±0.7 in RA and −1.0±0.5 in osteoarthritis *versus* −1.8±0.2°C, p<0.001). The 5 RA knees with power Doppler findings indicative of grade 2 inflammation had a ΔΤ 3 times lower compared to healthy (−0.6±0.6, p = 0.007), whereas the 9 RA and the 7 osteoarthritis knees with additionally fluid effusion, had even lower ΔΤ (−0.4±0.7, p<0.001).

**Conclusion:**

Using a safe, rapid and easy-to-perform method, such as microwave radiometry, thermal changes within the knee joint may reflect non-clinically apparent joint inflammation. Refinement of this method, including production of sensors for small joints, could result to the development of the ideal objective tool to detect subclinical synovitis in clinical practice.

## Introduction

Rheumatoid arthritis, the most common systemic autoimmune disease, is characterised by the chronic inflammation in multiple joints which leads to cartilage damage and bone erosion. Joint inflammation is associated with peri-articular vasodilatation and synovial proliferation, which is accompanied by intra-articular angiogenesis [1]. Synovitis is not only the central characteristic of the pathophysiology of rheumatoid arthritis (RA), but also a key feature of osteoarthritis (OA) which affects hundreds of millions of people worldwide [2]. Since the activated synovium may produce proteases and cytokines that accelerate structural damage, the early and accurate detection of synovial inflammation, often localized and sometimes asymptomatic, is important [2].

Synovitis has traditionally been assessed by means of clinical and laboratory parameters suggestive of local inflammation. Modern imaging techniques, such as musculoskeletal ultrasound and magnetic resonance imaging (MRI), are playing an increasingly important role in the evaluation and monitoring of arthritis activity [3], [4]. Use of ultrasound with power Doppler, a routinely available bedside imaging method, has become more sensitive and reproducible than clinical evaluation in assessing increased synovial vascularization in patients with arthritis, particularly in the early stages when X-rays are normal [1], [4]. MRI, especially contrast-enhanced, has also proven to be more sensitive and reliable than clinical examination in the detection of synovial inflammatory activity and has the ability to quantify changes in synovial volumes and erosions [4]. At present, MRI involves substantial time and cost, exposure to contrast agents, and is not widely available for routine clinical use in many countries. Importantly, both ultrasound and MRI can detect subclinical synovial inflammation’s presence which may explain the structural progression reported in RA patients who satisfy conventional criteria of disease remission [5].

Microwave radiometry (MR) detects non-invasively and accurately the relative changes of temperature in human tissues in depth [6], [7]. MR measures natural electromagnetic radiation from internal tissues at microwave frequencies, based on the principle that the intensity of the radiation is proportional to the temperature of the tissue. Thermographic detection of human cancers by MR has been tried in early studies, albeit without considerable clinical implications [6]. Most recently, the safety and the effectiveness of MR to detect subclinical local inflammatory activation has been demonstrated in carotid atherosclerotic plaques [8], [9]. No method is currently being employed for the *in vivo* measurement of joint temperature as an indicator of synovitis.

In initial studies we confirmed that the obtained *in vivo* temperature measurements by MR from joints with clinically overt inflammation was highly increased in patients with knee, ankle and carpal monoarthritis, when compared to the non-inflamed symmetrical joint. Therefore, the present proof-of-concept study was conducted to test the hypothesis that, in the absence of relevant clinical signs, MR may detect increased local temperature reflecting subclinical synovial inflammation which is, in parallel, diagnosed by joint ultrasound.

## Patients and Methods

### Ethics Statement

The study was approved by the Ethical/Scientific Committee of Laikon University Hospital and all subjects- patients and healthy controls- provided written informed consent according to the Declaration of Helsinki.

### Protocol

Consecutive non-febrile patients with either RA (n = 20, aged 57.2±10.5 years) or OA (n = 10, aged 61.9±6.5 years) who fulfilled the respective American College of Rheumatology classification criteria were examined. In addition to their willingness to participate in the study, the only inclusion criterion was the absence of pain and any clinical sign of knee joint inflammation in physical examination performed by a rheumatologist. Healthy subjects without a history of knee trauma (n = 15, aged 32.1±6.9 years) served as controls. To further elaborate the hypothesis that increased local temperature detected by MR reflects subclinical synovial inflammation we also examined control subjects who had non-inflammatory unilateral knee pathology, either meniscal tear or cruciate ligament injury confirmed by MRI, in the past 3 months (n = 10, aged 45.6±6.5) All underwent evaluation by MR, followed immediately by knee ultrasound by an experienced operator who was blinded to MR measurements.

### Microwave Radiometry Measurements

The MR measurements were performed with the RTM 01 RES microwave computer based system (Bolton, UK) that detects temperature from internal tissues at microwave frequencies. The basic principles of MR have been previously described [7]. Briefly, the essential basis for developing a microwave imaging technique is the significant contrast in the dielectric properties, at microwave frequencies, of different tissues. The depth of penetration depends on the wavelength, the dielectric properties and the water content of the tissue. The system of MR possesses an antenna with two sensors: a microwave and an infrared. The microwave sensor is 3.9 cm in diameter and detects microwave radiation at 2–5 GHz spectrum, which corresponds up to 7 cm in depth, with accuracy for temperature measurements of ±0.2°C. The sensor blocks all microwave signals from the environment. The ‘volume under investigation’ is a rectangular area of 3 cm in width, 2 cm in length, and 3–7 cm in depth depending on the dielectric properties of the underlying tissues. The second sensor is used for infrared measurements from the skin, for calibrating the microwave sensor readings [8], [9].

**Figure 1:**
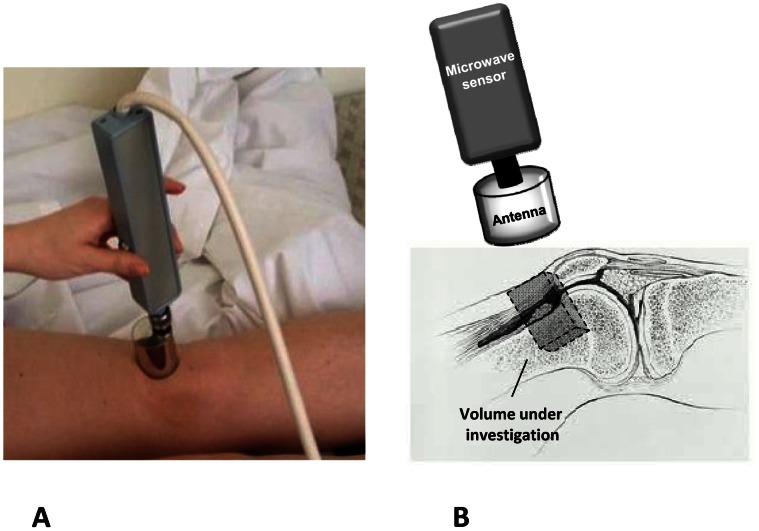
The microwave radiometry system. (A) The antenna for microwave radiometry device placed at 90° angle over the knee joint. (B) Schematic presentation of the system of microwave radiometry. The antenna of the microwave sensor is in contact to the skin above the volume under investigation, i.e. the knee joint.R.

All subjects were examined in a room with steady temperature (21–23°C) and before the temperature reading procedure they rested for 10 minutes to equilibrate to the ambient temperature. During measurements, with the individual in supine position, the microwave antenna of the device was placed in contact with the naked skin at 90° angle. The antenna was held at this position for 10 sec, required for the receiver to integrate the microwave emission and the conversion of the measured signal to temperature by a microprocessor. Measurements were taken at the upper one third of the anterior surface of the thigh, which was used as control point, and on the upper pole of the patella, by placing the antenna at the site over the suprapatellar recess **(** **Figure 1** **)**. All measurements were performed 3 times at each of the 2 sites for every leg, in order to assess the reproducibility of the method (overall 12 measurements for each subject by each of 2 operators). The temperature of each site used for further analysis was the mean of the 3 temperatures. As the temperature of the knee was different than the temperature of the thigh, temperature differences (ΔT) were provided rather than absolute temperature values. ΔT for each leg was defined as the knee joint temperature (suprapatellar recess) minus the thigh temperature (control point). The mean value of the two ΔTs calculated by the two different operators was used for the analysis.

### Ultrasound Imaging

Knee joint ultrasound was performed after the completion of MR measurements, to ensure that the water-based gel used for ultrasound will not influence the temperature distribution and the readings. All ultrasound assessments were performed using a 8–13 MHz linear transducer (Logiq P3, GE) by an experienced sonographer who was blinded to the subject’s history and temperature readings. The scans were based on a protocol derived from EULAR guidelines [10] while the OMERACT guidelines for synovial effusion [11] were also met. Synovial effusion in grayscale was defined as an abnormal anechoic or hypoechoic area in the joint that is displaceable and compressible and lacks Doppler signal; as per the OMERACT guidelines [11].

The size of effusions was measured in the longitudinal suprapatellar position, with the knee in 30° of flexion. The maximum diameter of the effusion in the longitudinal view was used to quantify it and increased fluid in the suprapatellar recess was defined as >2.4 mm [10]. Synovial blood flow was evaluated by power Doppler ultrasound in the knee joint. Active synovitis was defined as the presence of intra-articular synovitis with power Doppler signal, which was graded on a semiquantitative scale from 0 to 3 (0 =  absence, no intra-articular flow; 1 =  mild, single-vessel signal or isolated signals; 2 =  moderate, confluent vessels; 3 =  marked, vessel signals in more than half of the intra-articular area) during the ultrasound examination [12].

### Statistical Analysis

Statistical analysis was performed using Mann-Whitney U and significance was defined as p<0.05. Results are presented as the mean ± standard deviation.

## Results

In the 15 healthy control subjects examined the obtained knee temperature by MR was always lower than thigh (32.3±1.1 *versus* 34.1±0.9°C, p<0.001 respectively), with a ΔΤ of −1.8±0.2°C. As expected, there were no abnormal findings in ultrasound examination. In the 10 control subjects with recent history of knee trauma, the obtained temperature from the injured knees was lower than the ipsilateral thigh (31.8±1.4 *versus* 33.6±1.2°C, p<0.001), with a ΔΤ of −1.9±0.2°C (p = 0.368 *versus* healthy) and similar to the contralateral non-injured knee (32.3±0.7, p = 0.273). Mild fluid effusion (2.6±0.3 mm) was detected in 6 knees, while power Doppler signal was not detected by ultrasound in any of the injured knees.

**Figure 2:**
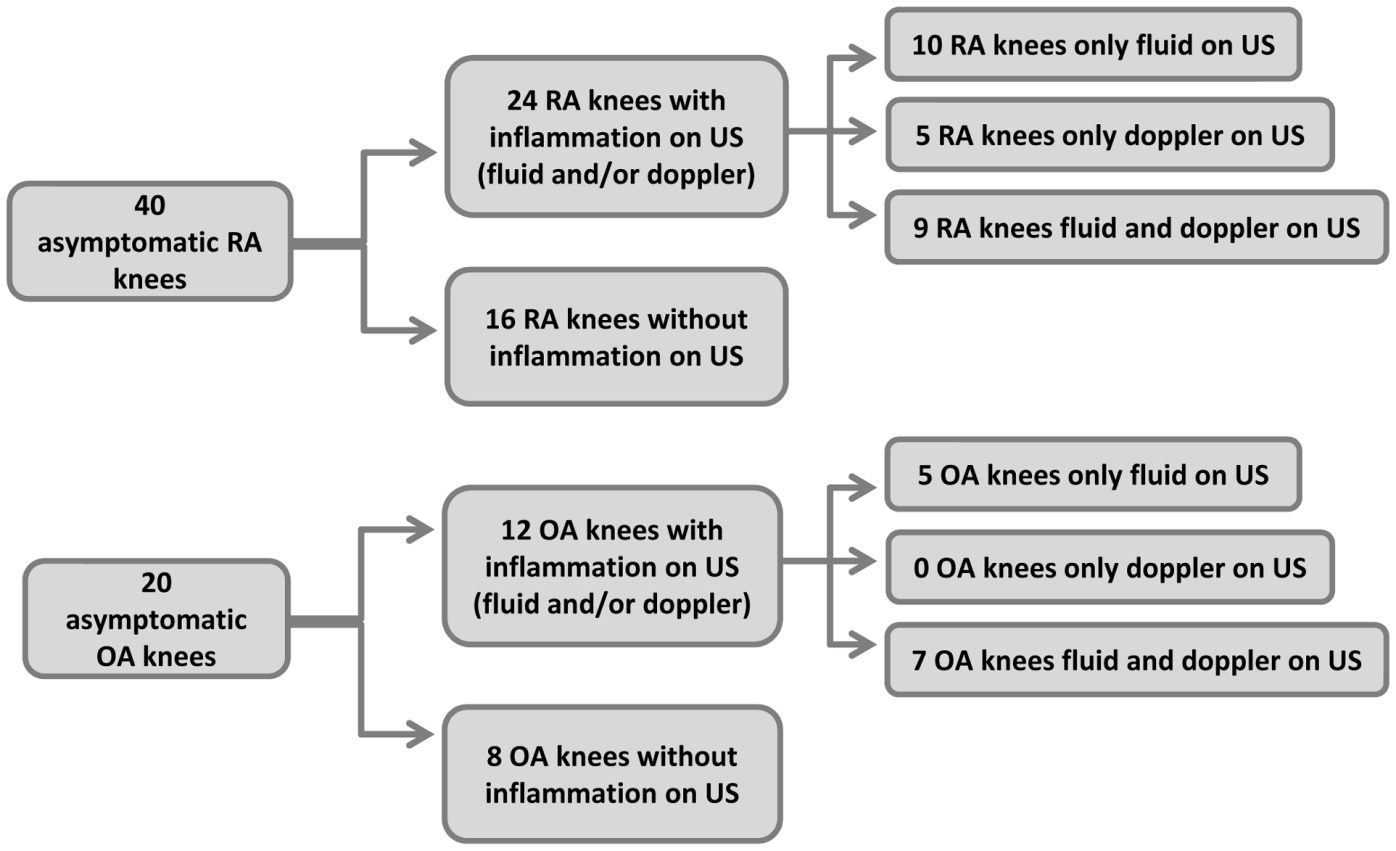
Subgroups of patients. Flow chart showing rheumatoid arthritis (RA) and osteoarthritis (OA) patients who were examined by microwave radiometry and the resulting subgroups based on findings indicative of subclinical inflammation in at least one knee, by ultrasound (US).

Forty RA and 20 OA asymptomatic knees were then examined. As shown in **Figure 2**, based on the ultrasonographic findings indicative of subclinical inflammation in at least one knee, the patients were further divided into subgroups. MR temperature readings of the thigh (control point) were similar to healthy subjects and comparable in all subgroups irrespectively of knee ultrasound findings. Namely, in the RA (N = 24) and OA (N = 12) knees with ultrasound findings indicative of subclinical inflammation the thigh temperature was 34.2±0.8°C (p = 0.969) and 34.1±0.9°C (p = 0.897) *versus* control thighs respectively. In addition, in the 16 RA and 8 OA knees without ultrasound findings indicative of subclinical inflammation the thigh temperature was 33.7±0.9°C (p = 0.418) and 34.1±0.8°C (p = 0.904) *versus* healthy thighs respectively. Asymptomatic RA and OA patients had comparable Erythrocyte Sedimentation Rate at 1h, whether or not knee ultrasound examination was positive for fluid and/or synovitis (*data not shown*).

**Figure 3:**
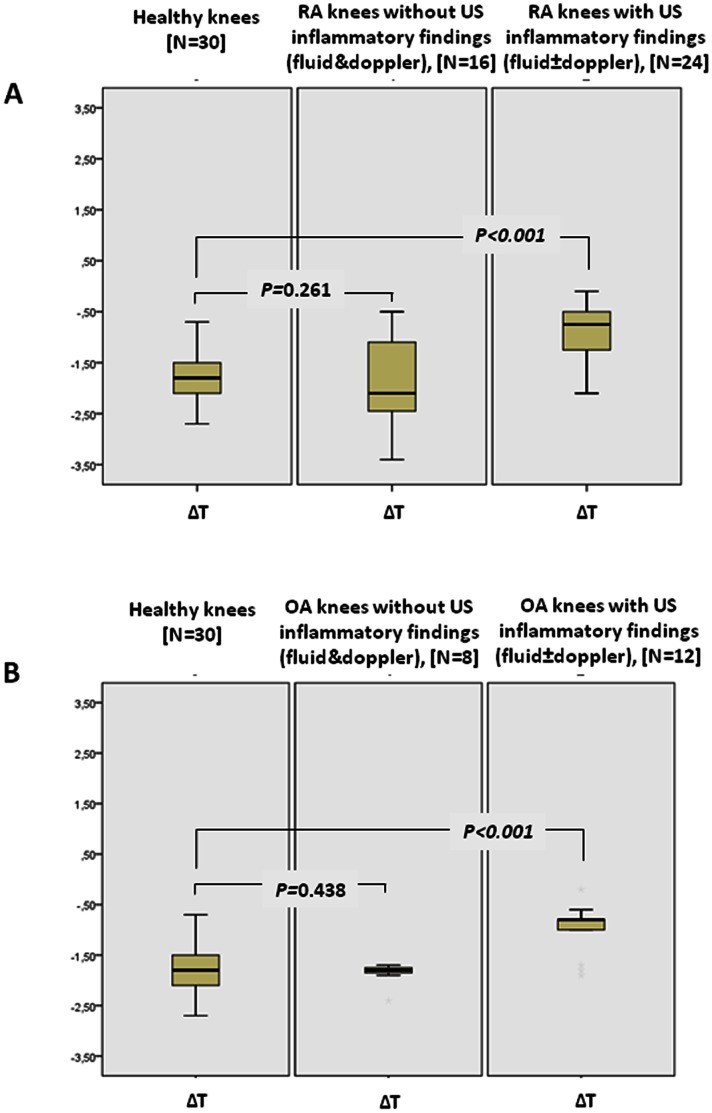
Temperature differences (ΔT) recorded by microwave radiometry device. Temperature differences (ΔT) between knee joint and thigh (control point) of healthy subjects and (A) patients with rheumatoid arthritis (RA) or (B) patients with osteoarthritis (OA) stratified by the presence of excess fluid and/or Doppler signal evidenced by knee joint ultrasound (US). Patients’ knee joints with inflammatory US findings had smaller ΔT compared to healthy knees and patients’ knees with normal US findings.

In RA knees with ultrasound findings indicative of subclinical inflammation (fluid effusion and/or Doppler signal, N = 24) the temperature was higher than normal knees (33.1±1.0 *versus* 32.3±1.1°C, p = 0.058) and the ΔΤ significantly lower (−0.9±0.7 *versus* −1.8±0.2°C, p<0.001), (**Figure 3Α**). Similarly, OA knees with ultrasound findings indicative of inflammation (fluid effusion and/or Doppler signal, N = 12) had a significantly lower ΔΤ (−1.0±0.5 *versus* −1.8±0.2°C, p<0.001), (**Figure 3B**).

Intra-articular synovitis with power Doppler findings indicative of grade 2 inflammation was evident in 5 of 40 RA knees, 9 had additionally fluid effusion, while 10 had only fluid effusion. In these 5 knees the ΔΤ was 3 times lower compared to healthy (−0.6±0.6 *versus* −1.8±0.2°C, p = 0.007), in the 9 knees the ΔΤ was even lower (−0.4±0.7, p<0.001), while in the 10 RA knees the ΔΤ was not significantly different than healthy knees (−1.6±0.7°C). Comparable results were found in 7 OA knees with power Doppler and fluid (ΔΤ = −0.6±0.8, p<0.001 *versus* healthy knees) and in 5 OA knees with only fluid (ΔΤ = −1.3±1.7) (**Table S1**). The mean size of the maximum diameter of effusion measured in the longitudinal view with the knee in 30° flexion was 2.8±0.3 mm in the RA group and 3.0±0.4 mm in the OA group. Intraobserver and interobserver differences of MR measurements were minimal (0.06±0.08°C and 0.08±0.11°C, respectively).

## Discussion

In this proof-of-concept study the knee joint was elected for technical reasons related to the shape and the size of the currently available MR device. In patients with arthritis of inflammatory origin (RA or OA), compared to healthy controls and post-traumatic control knees, we found that: a) thermal changes within an asymptomatic knee joint can be indeed detected by MR, a safe, rapid and easy-to-perform, objective method b) higher temperatures detected by MR in RA or OA knees correlated with findings indicative of joint inflammation by ultrasound, used as reference method. Although, a limitation of the currently available MR technique is that it cannot clearly differentiate inflammatory arthritis of different aetiology and does not directly quantify functional or structural damage, this method appears to be an indirect indicator of joint inflammation, reflecting increased local (neo)vasculature content and low level inflammation which is not translated in clinical signs.

Swelling and temperature changes estimated during assessment of the number of joints with active arthritis are essential to determine inflammatory status in clinical practice. Imaging techniques (ultrasound, contrast-enhanced MRI) are increasingly used to improve the assessment of arthritis activity. On the other hand, radioisotopic and thermographic methods have been explored to quantify active arthritis with variable results. In addition to the fact that radioisotope use is a major limitation, correlations between thermographic indices and clinical assessment results have been conflicting [13]. Infrared thermography studies have shown that joints have normal thermal patterns based on the principle that a negative temperature gradient exists from the centre of a normal joint to the skin. The heat generated by increased vascularity in synovial inflammation distorts this gradient, and the normal thermal pattern is lost [14]. More recently, the ability of three-dimensional and thermal surface imaging to produce measures of joint volume, shape, and temperature to aid in the assessment of disease activity in arthritis has been reported, albeit with limitations related to joint deformities, when present, and with questionable inter- and intra-user variability [15]. MR can detect natural electromagnetic radiation from internal tissues at microwave frequencies, and the intensity of radiation is proportional to the temperature of the tissue. The main difference between the previously used infrared thermography and MR is that the former is a method of superficial screening, has only a very limited depth of penetration- approximately 2 mm- and thus allows to read and display skin temperature, whereas the latter detects signals at a much greater depth, up to 7 cm, reflecting the internal tissue temperature [6]–[9]. The possible impact of other adjacent tissues on the measurements of temperature in the current study seems limited, as the knees of patients without actual inflammation in ultrasound had comparable ΔΤ to healthy knees. It is known that although a positive power Doppler signal is indicative of active inflammation, negative signal does not necessarily mean absence of active synovitis [16]. Yet, as most recently shown, smoldering inflammation reflected by positive synovial vascularity (power Doppler) under low disease activity is linked to joint damage in RA [17]. Therefore, monitoring of synovial vascularity, as reflected by increased MR measurements, has the potential to provide useful joint information to tailor treatment strategies.

To conclude, the findings presented herein reinforce the utility of additional to physical examination methods for the accurate evaluation of disease status in patients with arthritis. MR may be potentially used to identify patients who might benefit from closer monitoring, early intervention, or more aggressive therapy. The combined prognostic value of MR with an imaging technique needs to be studied. Since thermal changes cannot be sensed by skin palpation in every joint, whereas they may precede inflammatory changes that can be detected by imaging methods, it is a challenge to refine MR methods and produce sensors for smaller joints that could detect increased temperature in precise and/or targeted depths (i.e. specifically at synovium). This method may also provide additional information on the natural history of chronic arthritis. Prospective studies to reveal whether MR can serve as an objective tool to detect early and/or subclinical synovitis in the clinical setting, as well as its possible prognostic value are warranted.

## Supporting Information

Table S1 **Microwave radiometry recordings for all patients, stratified according to the knee ultrasound findings.** Mean value ± standard deviation of absolute temperatures (T) and difference in temperature (ΔT) are given.
(DOC)
