# Supplementary material for: Detection of Subclinical Synovial Inflammation by Microwave Radiometry
Source: PLoS One. 2013 May 31;8(5):e64606. doi: 10.1371/journal.pone.0064606 (PMC3669424; doi:10.1371/journal.pone.0064606)
Supplement: Table S1 — Microwave radiometry recordings for all patients, stratified according to the knee ultrasound findings. Mean value ± standard deviation of absolute temperatures (T) and difference in temperature (ΔT) are given. (DOC) [file pone.0064606.s001.doc]

**Table S1**. Microwave radiometry recordings for all patients, stratified according to the knee ultrasound findings. Mean value ± standard deviation of absolute temperatures (T) and difference in temperature (ΔT) are given.

|  |  | **Thigh T (°C)** | **Knee T (°C)** | **ΔT (°C)** |  | **Thigh T (°C)** | **Knee T (°C)** | **ΔT (°C)** |
| --- | --- | --- | --- | --- | --- | --- | --- | --- |
| **40 asymptomatic RA knees** | 24 knees with US indicative of inflammation | 34.2±0.8 | 33.1±1.0 | -0.9±0.7 | only fluid, n=10 | 34.1±0.9 | 32.5±0.5 | -1.6±0.7 |
|  |  |  |  |  | only Doppler sign, n=5 | 34.5±0.9 | 33.9±0.8 | -0.6±0.6 |
|  |  |  |  |  | both fluid & Doppler sign, n=9 | 34.4±0.7 | 34.0±0.5 | -0.4±0.7 |
|  | 16 knees without US evidence of inflammation | 33.7±0.9 | 32.6±1.6 | -1.2±1.2 |  | | | |
| **20 asymptomatic OA knees** | 12 knees with US indicative of inflammation | 34.1±0.9 | 33.2±0.9 | -1.0±0.5 | only fluid, n=5 | 34.1±0.9 | 32.7±1.0 | -1.3±1.7 |
|  |  |  |  |  | both fluid & Doppler sign, n=7 | 34.3±0.6 | 33.6±0.9 | -0.6±0.8 |
|  | 8 knees without US evidence of inflammation | 34.1±0.8 | 32.4±0.5 | -1.7±0.4 |  | | | |

RA: rheumatoid arthritis; US: ultrasound; OA: osteoarthritis.
